# Some Observations on Diphtheria

**Published:** 1917-06

**Authors:** A. D. Pattillo

**Affiliations:** Wichita Falls, Texas


### Some Observations on Diphtheria.

BY DR. A. D. PATTILLO, WICHITA FALLS, TEXAS,

I shall not attempt to cover this subject in detail, but merely want to discuss some points which I think might be discussed to the advantage of the profession.

The diagnosis of the dissease is usually, though not always, easy. The patient usually complains of sore throat, painful deglutition, malaise. The child usually lies around the house, not caring for its usual sports, though does not appear sick to the casual observer. There is usually hoarseness after a few hours, or a day or two at least, and difficulty of breathing. There is frequently swelling of the throat, tonsils and lymphatic glands, to such an extent as to be visible externally. In the majority of cases there is seen on the faucial tonsils, uvula, or soft palate, a dirty, grayish white membrane consisting of diphtheria bacilli and inflammatory exudate.

The temperature runs from 99 to 101 or 102, but never as high as in acute tonsilitis. The pulse may not be materially altered at first, but later becomes rapid and weak, as the disease progresses. There is rapid and labored breathing, accompanied by asphyxiation, which bring up the tail end of the train of symptoms, as seen late in the disease, and end in death in all cases not treated early, rationally and radically.

If there be any doubt as regard diagnosis, it may be cleared up by microscopic examination of the exudate.

Have on hand some tubes of horse serum, hermetically sealed, so that it does not dry out; take a swab from the throat, inoculate the tube of horse serum, put it in your inside pocket (if you have no thermostat), carry it there for six to twelve hours and you have a growth of a dirty grayish, or brownish color; take a platinum loop, transfer some of this to a slide, fix, stain with Loeffler's blue for one-half to one minute, and examine under 1-12 oil immersion lens; you have myriads of club-shaped bacilli, with a beaded appearance, owing to alternating light and darkly staining areas. This obviates the time-consuming process of sending swabs many miles to a bacteriologic laboratory.

However, if the aforesaid symptoms become alarming, and the disease is progressing rapidly, one should not wait for a microscopic examination, but begin treatment at once with large doses of antitoxin.

Where a child has croup one night, not abating but continuing through the next day, it should be regarded as diphtheria, even though no membrane is visible, and, if it continues, the child should be given antitoxin, just as in a case where the membrane is visible, for it is, in all probability, suffering from a case of laryngeal diphtheria.

*Prophylaxis.*—As regards prophylaxis, of course, it goes without saying that the patient should be isolated, and strict asepsis, and antisepsis covering everything which comes in contact with the patient or his room, should be observed.

I would like to say, before I go further, that I think it unnecessary expense, inconvenience and pain, to administer antitoxin to children who can be seen by a physician every day. Some children are already immune, and those who are not may be not sufficiently exposed to contract the disease. Furthermore, the disease is not very highly contagious or infectious. I take this view because of the pain and expense which it entails. Also the anaphylaxis which sometimes develops after the use of the horse serum. Furthermore, the immunity which they gain from a small dose of antitoxin is only passive, and will last only a few weeks, or at most a few months. They may subsequently contract the disease, and, of necessity, require many doses of the antitoxin; and, if they have previously been given antitoxin, they may develop symptoms due to anaphylaxis against horse serum.

A brief mention of a personal experience will show how beautifully this was demonstrated to the writer. Last winter while doing a tracheotomy on a child who was dying from the disease in question, I contracted diphtheria myself. Just one year prior to this I had an attack, contracted in the same way. During the first attack I received 25,000 units of antitoxin. The result was when I received the serum for the second attack I developed a general edema over the body, with a marked reaction and edema at the site of injection (which in the case of myself was in the anterior abdominal wall). My abdominal wall, scrotum and penis and thighs even down to my knees looked like a patient suffering from a general anasarca; and the itching is not to be described by the word *"itch."*

There is now in use what is known as the Shick test, for immunity to diphtheria. The most extensive discussion of the subject which I have seen is contained in the January number of the *Medical Clinics of Chicago,* reference to which is made for a fuller discussion of the test. The test consists of a solution of the products of diphtheria bacilli. The minimum lethal dose of this toxin for a 250-gram guinea pig is determined, and about one-fiftieth of this amount is used to make the test in man. The dose used is about one-tenth of a c.c. or about 1½ minims. The injection is made intracutaneously with a finely graduated syringe, similar to the method used in making the tuberculin test. It produces a reaction, consisting of redness and swelling at the site of injection, if the patient is susceptible to diphtheria. If there is no reaction, the patient is immune to diphtheria, and need not be subjected to the expense, pain and inconvenience of antitoxin.

This test is of value where children have been exposed to the disease, those who are immune sought out, and those who are not immune observed more closely. For, as I have already said, I do not think it necessary to adminster serum to children who have been exposed to this disease probably in a very light way, unless they be where they cannot be seen and examined at frequent intervals by a physician, for it only immunizes them for a brief period, and renders them liable to anaphylaxis to horse serum should they subsequently become infected with the diphtheria bacilli, which reaction is very unpleasant, inconvenient, and may be even dangerous.

*Treatment.*—In the treatment of this disease, I want to say that I do not believe that anything less than a 5000-unit dose has any place in the physician's armamentarium. And I much prefer the 10,000-unit dose in cases where the patient is three or four years old. The case cannot be cured with less than 10,000 units, and the larger doses you give, the sooner your case will recover, and the less antitoxin will be required.

Any other treatment more than stimulation, care of the bowels, kidneys and diet is not worthy of consideration. Remedies directed toward dissolving the membrane, or disinfecting the throat, etc., serve only to annoy and disturb the patient. Nothing dissolves the membrane like plenty of antitoxin.

The best mode of administration is that which will bring the serum into the blood stream the quickest, and is, of course, intravenously. This method is difficult in children with small veins, and with a great amount of adipose tissue—also late in the disease where the heart has become so weak that it will not distend the vein, after limb has been corded.

The next best way is by injecting intramuscularly, absorption taking place much more rapidly than when injected subcutaneously, as shown by recent experiment.

I now come to the burden of my paper: "The treatment of these cases after antitoxin has failed, and the patient is doomed to die." When the patient begins to toss about the bed, turn the head from side to side, throw its arms and pull at the cover; the face is blue; the pupils dilated; the pulse rapid and weak; you know that a very few hours, perhaps minutes, will close the scene.

Are you going to stand by and say, "The Lord's will be done"? For humanity's sake, if there is not an experienced physician or surgeon near who can do intubation, you should have on hand a knife, scissors, artery forceps and a tracheotomy tube; open the trachea and give the child oxygen till nature and the antitoxin can handle the situation.

I wish to report briefly some cases which have come under my observation recently, and have a bearing on this subject.

Case 1.—A boy aged 6; well nourished and previously healthy. I first saw the boy on Saturday night suffering from croup, of about twenty-four hours' duration. I tried to examine his throat, but owing to a bad light I had a very unsatisfactory examination, and could see no membrane. I left instructions to call me next day, or let me hear from the child. This was done, and the child reported better. The next day, which was Monday, and about forty hours after my first visit, I was again called, and on examination found a well-developed membrane on both tonsils. I at once gave 5000 units of antitoxin, repeated the dose in eight hours and again in about the same length of time, giving 7500 units. The serum seemed to have no effect whatever, and the next morning, which was Tuesday, the fourth day of the disease, the child was rapidly growing worse, and I saw it must surely die unless something more radical was done. I advised tracheotomy, and was told to go ahead. I did a tracheotomy. As usual, respiration ceased, when the trachea was manipulated. Artificial respiration was begun immediately I had the tube in place, and after several minutes the respiratory rhythm was reestablished. The child made a good recovery, I have always thought that larger doses of antitoxin would have saved this child the necessity of tracheotomy

Case 2.—Girl, aged 6; well nourished and otherwise healthy. Called another physician on Sunday, 12 o'clock noon. He placed her on treatment for ordinary tonsilitis. The child grew rapidly worse till Tuesday noon, forty-eight hours after her condition was first noticed. At this time I was called and found the child dying from diphtheria. I advised a tracheotomy, which was concurred in by both the family and the physician in charge. I did the operation immediately, and in a very few minutes had the tube in the trachea. As usual, respiration ceased when manipulation of the trachea was begun. The patient died in spite of vigorous artificial respiration for several minutes.

CASE 3.—This case represents the laryngeal type, the one most likely to reach serious proportions before it is recognized. The patient, a boy, aged 4, well nourished and otherwise healthy, became somewhat indisposed on Monday. I was called on Wednesday, found the child somewhat croupy, temperature 99.6, pulse somewhat accelerated. It at once suspected diphtheria, but examination revealed only some enlarged tonsils. No membrane was visible. I advised the use of antitoxin in the belief that the child was suffering from laryngeal diphtheria, but the mother went frantic at the suggestion of the serum. No antitoxin was given at this time. The child seemed somewhat improved on the following day, and I saw him no more till Saturday, two days later. Patient had grown worse, his respiration was difficult, labored, croupous, and could be heard in all parts of the house. The child could not lie still, tossing about the bed and otherwise showing his air hunger. I advised the use of antitoxin immediately, if the family desired my further services. This was agreed to, and I gave the child 10,000 units at 8 p. m. The following morning, about twelve hours later, 10,000 units more were given. The condition cleared up immediately, and the child made a rapid recovery without further trouble or treatment, showing that it was a case of laryngeal diphtheria.

CASE 4.—Girl, 4 years old, well nourished and otherwise healthy, took sick of croup, after a day or two of prodromal symptoms. She rather suddenly became very croupous. Another physician was called who examined the patient and finding no membrane present told the family that the child did not have diphtheria, but saw something had to be done immediately. I was called to come at once and bring my instruments, as the child was choking to death. Upon my arrival, I found the child as limp as a dish rag, asphyxiated and moribund. I at once did a tracheotomy and administered antitoxin. The child made an uneventful recovery. This was a case of laryngeal diphtheria which had eluded the family as well as the family physician and would certainly have died without tracheotomy.

CASE 5.—Baby girl, aged 4 years; fairly well nourished; hygienic surroundings bad, previous history not known.

I was called eight miles in the country to see this child on the night following the seance of the previous case. I suspected diphtheria, and went out as fast as I could, carrying some antitoxin. Upon my arrival, I found a child dying of laryngeal diphtheria. Nothing approaching a membrane to be seen in the throat. I asked why they had not had a physician sooner, and was told by the father that they did not think the child very sick, as the other children had had the same thing for several days. I told them that the child was dying of laryngeal diphtheria, and would not live thirty minutes. I tried to administer a 10,000-unit dose of antitoxin intravenously, but the child was so near dead that the heart would not distend the vein when the arm was corded. I could not give the serum on account of a faulty syringe, nor would it have done any good if I had. There was only one thing left to do and that was tracheotomy. I took the child in my car and made a mad rush for my office eight miles away; telephoned two physicians in town to meet me at my office. I reached the office before they did. I had used the only tracheotomy tube I had the day before, so I made one by sawing off the lower 2½ inches of a metal catheter with a gigle saw. I cut two cartilaginous rings, inserted the piece of catheter with adhesive strips wrapped tightly around the upper end to prevent its slipping into the trachea, and resuscitated the child by artificial respiration. In a few seconds the child drew a deep inspiration and opened its eyes with a look of delight and began trying to call for water.

The child lived, took nourishment and water for eighteen hours, when she became choked up and died.

Out of four cases operated on, I had a mortality of 50 per cent, as against 100 per cent if they had not been operated on; and three of these were not seen until they were breathing their last.

---

The General Medical Board of the Council of National Defense has arranged to send 3000 surgeons to the front within the next three months, anticipating the consent of the government, which may be assumed.

